# Do women with prior obstetrical anal sphincter injury regret having a subsequent vaginal delivery?

**DOI:** 10.1186/s12884-019-2380-x

**Published:** 2019-07-04

**Authors:** Madeline Edwards, Emily K. Kobernik, Shriya Suresh, Carolyn W. Swenson

**Affiliations:** Department of Obstetrics and Gynecology, University of Michigan, 1500 E. Medical Center Dr, Ann Arbor, MI 48109 USA

**Keywords:** Obstetrical anal sphincter laceration, Third-degree perineal laceration, Fourth-degree perineal laceration, Regret, Satisfaction, Vaginal delivery

## Abstract

**Background:**

Most women choose to have another vaginal delivery following one complicated by an obstetrical anal sphincter injury (OASIS). However, little is known about patient satisfaction or regret with this decision. Therefore, our objective was to assess decisional regret with subsequent route of delivery following one affected by an OASIS.

**Methods:**

A survey study was conducted among women seen in a specialty postpartum perineal clinic at a tertiary teaching hospital following a vaginal delivery with an OASIS between March 2012 and December 2016 who also had a subsequent delivery during that time period. Women were mailed a 13-item questionnaire between June and October 2017 that addressed pelvic floor symptoms and regret with their decision regarding mode of subsequent delivery. Regret was assessed with a modified Decision Regret Scale. Bivariate analyses were used to compare women with no, mild, or moderate/severe regret.

**Results:**

Among 115 eligible women, 50 completed the survey. The majority (82%, *n* = 41) had a subsequent vaginal delivery and 18% (*n* = 9) had a subsequent cesarean delivery. Over one-third (34.9%, *n* = 15) reported the counseling they received after the OASIS influenced their decision regarding route of subsequent delivery. Fifty-four percent (*n* = 27) had no regret regarding their decision about subsequent delivery route, while 18 (36%) had mild, and five (10%) had moderate/severe regret. Regret was associated with older age (none: 36.8 ± 3.6 vs mild: 37.3 ± 3.4 vs moderate/severe: 41.7 ± 3.8 years, *p* = .03) and prevalence of fecal incontinence after delivery with OASIS (none: 15% vs mild: 17% vs moderate/severe: 80%, *p* = .01).

**Conclusions:**

Most women with an OASIS and a subsequent pregnancy will choose a repeat vaginal delivery, and over half have no regret about this decision. Older age and fecal incontinence following the incident delivery with OASIS were associated with regret regarding subsequent delivery mode.

## Background

In 2018, The American College of Obstetricians and Gynecologists published new guidelines for optimizing postpartum care [1]. In this committee opinion, they emphasize the importance of postpartum counseling regarding any complications that occurred during pregnancy or delivery and implications of those complications for future pregnancies. Obstetrical anal sphincter injury (OASIS), including both third- and fourth-degree lacerations, complicates up to 18% of primary vaginal deliveries (VD) [2] and has a recurrence rate of up to 9% [3–5]. OASIS is associated with significant morbidity including perineal pain, urinary incontinence, anal incontinence, and sexual dysfunction [2]. Subsequent VD following a delivery with an OASIS has been shown to worsen pelvic floor symptoms [6]. Despite these data, 65% of women still choose to have another VD following one with an OASIS [3]. Patient satisfaction with this decision is unknown, which is a significant knowledge gap when it comes to counseling women on mode of delivery following an OASIS.

At our institution, we have a specialty postpartum clinic staffed by urogynecologists called Michigan Healthy Healing After Delivery (MHHAD) for women with, or at high risk for, pelvic floor disorders [7]. Approximately 50% of patients seen in MHHAD are referred for follow-up or complications of obstetrical lacerations. It is customary for most women who deliver at our institution and have an OASIS to be referred to the MHHAD clinic for follow-up within 2–4 weeks of delivery for evaluation, education, and counseling regarding their perineal laceration and future pregnancies.

Using the MHHAD clinic population, the primary aim of this study was to assess decisional regret with subsequent route of delivery following one complicated by an OASIS. As a secondary aim, we sought to describe the relationship between demographics, obstetrical factors, and pelvic floor symptoms with decisional regret regarding subsequent delivery mode.

## Methods

This was a mailed survey study that was conducted from June to October 2017. Women were included if all of the following criteria were met: 1) prior vaginal delivery complicated by OASIS (i.e., third- or fourth-degree perineal laceration), referred to as the “incident” delivery, 2) evaluation in the MHHAD clinic following the incident delivery, and 3) subsequent pregnancy and delivery (“subsequent” delivery)—all between March 2012 and December 2016.

Women were initially mailed a letter providing information about the study, including the option to complete the survey by phone or electronically; a written consent form, a copy of the survey; a prepaid return envelope; and study coordinator contact information. After 10 days, if the survey had not been returned and if the study team had not been notified by potential participants that they did not want to participate, women were contacted by telephone. Women were called a maximum of three times. Women received $5 in compensation for participating in the study. All participants provided written informed consent.

The 13-item questionnaire addressed basic knowledge of perineal lacerations, previous and current pelvic floor symptoms, satisfaction with clinic counseling received, and regret regarding route of subsequent delivery. Demographics, medical history (e.g., depression/anxiety, tobacco use, diabetes, and hypertension), pelvic floor symptoms (e.g., pelvic pain/dyspareunia, urinary incontinence, fecal incontinence, and pelvic organ prolapse), and delivery characteristics were collected via retrospective chart review. The primary outcome of interest was decisional regret, measured using the Decision Regret Scale (DRS), a validated tool that measures distress or remorse after a specific health care decision [8, 9]. Participants were asked to respond to statements regarding their subsequent route of delivery on a 5-point Likert scale from 1) “Strongly Disagree” to 5) “Strongly Agree.” Responses were converted to a range of 0–100, with higher scores indicating higher regret regarding subsequent route of delivery. The DRS also has three questions addressing satisfaction that are scored individually and have responses on a 5-point Likert scale.

Descriptive statistics such as frequencies, proportions, means, and medians were calculated for all variables. The distribution of decisional regret scores was observed by reviewing skew, kurtosis, QQ plot, histogram, and the Shapiro-Wilk test, and was found to be right-skewed. Decisional regret scores were categorized into three groups: none, score 0; mild, score 1–29; moderate/severe: ≥30 [10]. Comparisons of demographics, delivery characteristics, and survey responses across decisional regret groups were compared using Wilcoxon Rank test, Chi-square test, and Fisher’s exact test where appropriate. All statistical analyses were generated using SAS version 9.4 (SAS Institute, Inc., Cary, North Carolina). This study received approval from the Institutional Review Board at the University of Michigan (HUM00127908).

## Results

A total of 115 women were identified as eligible and mailed the questionnaire. Of these, 65 women did not respond or were excluded for incomplete data, leaving 50 women from whom data were collected and analyzed. Average age of respondents was 38.4 ± 3.8 years, 95.9% were Caucasian, median parity was 2, and average time since most recent delivery was 6.1 ± 2.4 years.

**Fig. 1:**
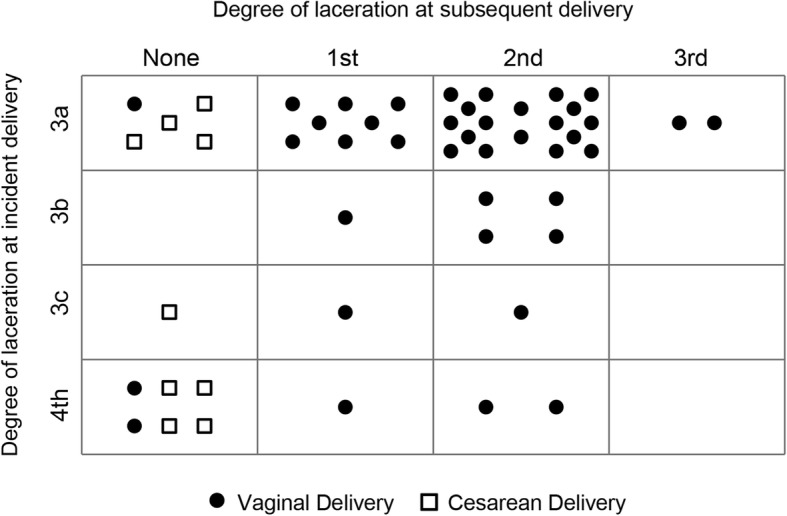
Trend of Perineal Lacerations with Incident to Subsequent Delivery. The vertical axis shows type of perineal laceration from the incident (OASIS) delivery and the horizontal axis shows the type of perineal laceration at the subsequent delivery. Each dot (subsequent vaginal delivery) or square (subsequent cesarean delivery) represents a single woman. For example, the upper left corner box shows that 5 women had a 3a laceration with their incident delivery and no lacerations with their subsequent delivery; 4/5 chose a cesarean for their subsequent delivery

All women had an OASIS with the incident delivery; 82% (41/50) had a third-degree laceration and 18% (9/50) had a fourth-degree laceration. For their subsequent delivery, 82% (*n* = 41) opted for a repeat VD, 16% (*n* = 8) had a planned cesarean delivery (CD), and 2% (*n* = 1) had an unplanned CD. All of the planned CDs were scheduled to minimize any additional pelvic floor injury. Figure 1 demonstrates the frequency of perineal laceration types in the incident and subsequent deliveries. Of the 41 women with a subsequent VD, 61% (25/41) had a second-degree laceration and 4.9% (2/41) had a recurrent OASIS. There were no repeat fourth-degree lacerations, although 4/9 women who had a fourth-degree laceration in the incident delivery opted for a CD for their subsequent delivery.

Responses to the Decision Regret Scale items are presented in Table 1. Overall, the average regret score regarding route of subsequent delivery was 10.7 ± 19.1, with 27 women having no regret (score of 0), 18 women having mild regret (14.2 ± 7.5), and five women reporting moderate/severe regret (56.0 ± 28.8). As expected, women with no regret about their decision had the highest satisfaction scores and those with moderate/severe regret had the lowest satisfaction scores. Demographics and clinical characteristics were then compared across groups to identify potential factors associated with regret (Table 2). Compared to women with no or mild regret, those with moderate/severe regret were significantly older and a greater proportion reported fecal incontinence after the incident delivery. Additionally, this group less frequently identified the correct description of a third- or fourth-degree perineal laceration compared to those with no or mild regret; however, this difference did not reach statistical significance (60% (3/5) vs 92.6% (25/27) vs 83.3% (15/18), respectively, *p* = .10). Four out of the five women with moderate/severe regret recalled receiving counseling in the MHHAD clinic regarding route of subsequent delivery and only one was counseled to have a cesarean section in a subsequent delivery; All five had a subsequent vaginal delivery, which was their desired mode of delivery. All of these women also reported they would recommend the MHHAD clinic for a friend who had an OASIS. Seven out of the eight women with a scheduled CD had no regret about their decision; the other woman had only mild regret.

**Table 1:** Responses to the Decisional Regret Scale regarding chosen mode of delivery following OASIS

Statement	No Regret (*n* = 27)	Mild Regret (*n* = 18)	Moderate/Severe Regret (*n* = 5)	Overall *p*-value^a^	None vs Mild *p*-value^b^	None vs Moderate/Severe *p*-value^b^	Mild vs Moderate/Severe *p*-value^b^
It was the right decision	1.0 ± 0.0	1.4 ± 0.5	3.0 ± 1.4	<.0001	0.004	0.03	0.06
I regret the choice that I made	1.0 ± 0.0	1.2 ± 0.4	2.6 ± 1.8	<.0001	0.08	0.12	0.15
I would go for the same choice again if I had to do it over again	1.0 ± 0.0	1.2 ± 1.0	2.8 ± 1.5	<.0001	0.01	0.05	0.06
The choice did me a lot of harm	1.0 ± 0.0	1.6 ± 0.7	3.8 ± 1.3	<.0001	0.002	0.009	<.0001
The decision was a wise one	1.0 ± 0.0	2.0 ± 1.0	4.0 ± 1.0	<.0001	0.0007	0.003	0.0009
The decision I made was the best possible for me personally	5.0 ± 0.0	4.6 ± 0.5	2.4 ± 1.3	<.0001	0.002	0.01	0.02
I am satisfied that my decision was consistent with my personal values	4.9 ± 0.5	4.5 ± 0.6	2.2 ± 1.1	<.0001	0.06	0.005	<.0001
I am satisfied that this was my decision to make	5.0 ± 0.2	4.5 ± 0.5	2.4 ± 1.3	<.0001	0.002	0.01	0.02

Data presented as mean ± SD

*P*-values determined using ^a^ANOVA and ^b^Tukey test

**Table 2:** Comparison of demographics, delivery characteristics, and pelvic floor symptoms by categories of regret regarding mode of delivery following OASIS

	Level of Regret			
	None *n* = 27	Mild *n* = 18	Moderate/Severe *n* = 5	*p*-value
Age, years	36.8 ± 3.6	37.3 ± 3.4	41.7 ± 3.8	0.03
White/Caucasian race	25 (96.2)	17 (94.4)	5 (100.0)	>.99
BMI, kg/m^2^	25.3 (22.5, 30.4)	24.6 (23.9, 30.1)	23.4 (23.1, 23.8)	0.62
Parity	2.0 (2.0, 3.0)	2.0 (2.0, 3.0)	2.0 (2.0, 2.0)	0.33
Tobacco use	5 (18.5)	3 (16.7)	1 (20.0)	>.99
Depression/anxiety	10 (37.0)	5 (27.8)	2 (40.0)	0.76
Characteristics of Incident Delivery with OASIS and Postpartum Symptoms				
Mode of delivery				0.56
Vaginal	19 (70.4)	12 (66.7)	5 (100.0)	
Forceps assisted	2 (7.4)	4 (22.2)	0 (0.0)	
Vacuum assisted	3 (11.1)	0 (0.0)	0 (0.0)	
Perineal tear				0.17
Third-degree	20 (74.1)	17 (94.4)	4 (80.0)	
Fourth-degree	7 (25.9)	1 (5.6)	1 (20.0)	
Birthweight, g	3495 (3135, 3710)	3603 (3290, 3950)	3835 (3760, 3860)	0.10
Urinary incontinence	20 (74.1)	12 (66.7)	4 (80.0)	0.90
Pain with intercourse > 6 months	8 (29.6)	6 (33.3)	1 (20.0)	>.99
Fecal incontinence	4 (14.8)	3 (16.7)	4 (80.0)	0.01
Michigan Healthy Healing After Delivery Clinic Visit				
Counseled about perineal tear	15 (79.0)	9 (64.3)	4 (80.0)	0.77
Counseled regarding mode of future deliveries	10 (52.6)	8 (57.1)	4 (80.0)	0.73
Would recommend to a friend with OASIS	15 (79.0)	9 (69.2)	5 (100.0)	0.87
Characteristics of Subsequent Delivery and Current Pelvic Floor Symptoms				
Mode of delivery				0.36
Vaginal	20 (74.1)	16 (88.9)	5 (100.0)	
Cesarean	7 (25.9)	2 (11.1)	0 (0.0)	
Birthweight, g	3403 (3190, 3655)	3510 (3330, 3910)	3750 (3315, 3830)	0.32
Urinary incontinence	11 (55.0)	4 (36.4)	2 (50.0)	0.71
Pain with intercourse	3 (37.5)	1 (16.7)	0 (0.0)	0.69
Fecal incontinence	1 (25.0)	2 (66.7)	2 (50.0)	0.77

Data reported as n (%), mean ± SD, or median (IQR)

The most common reported complication following OASIS was urinary incontinence in 72% of women (*n* = 36), followed by flatal incontinence in 36% of women (*n* = 18). Additionally, 30% of women reported dyspareunia (*n* = 15), 22% reported fecal incontinence (*n* = 11), 8% reported repair breakdown (*n* = 4), and 8% reported rectovaginal fistula (*n* = 4).

## Discussion

In this survey study of women seen in a specialty postpartum perineal clinic following VD complicated by an OASIS, over 80% chose a vaginal approach for their subsequent delivery. Regardless of mode of the subsequent delivery, 90% of women reported no or mild regret with their chosen delivery route. Older maternal age and fecal incontinence following the incident delivery were factors associated with increased regret in bivariate comparison.

Our findings are consistent with prior studies showing that the majority of women with a prior OASIS will have a subsequent VD [3]. However, our results extend the literature by showing that the vast majority of these women are satisfied with this decision. All women with moderate/severe regret had a subsequent VD and we found that increased maternal age and fecal incontinence following an OASIS were risk factors for regret regarding this decision. These findings may be useful in expanding antenatal counseling to include subjective outcomes in addition to objective ones for patients considering delivery routes after suffering from an OASIS.

While no specific decision aid is used in the MHHAD clinic, providers approach counseling using a shared decision-making framework, where the patient’s desired mode of future delivery is considered in the context of her individual medical circumstances, goals, and values. Prior studies have shown that shared decision-making improves both health outcomes and satisfaction with care [11–13]. In the current study of patients seen in the MHHAD clinic, the prevalence of moderate/severe decisional regret was low and most patients reported that the counseling they received impacted their decision regarding subsequent delivery. Interestingly, women with moderate/severe regret less frequently identified the correct description of a third- or fourth-degree perineal laceration than those with no/mild regret. This suggests an opportunity for improvement in counseling women following an OASIS to ensure they understand the diagnosis and implications for future pelvic floor health and subsequent deliveries.

This study is unique in its access to a small and understudied population of women who suffered from an OASIS, were counseled in a specialty clinic, and subsequently became pregnant and delivered another child. All of these women delivered both pregnancies and received their peripartum care and counseling at the same institution, creating a robust set of data. However, there are several important limitations to this study. Our response rate was 43%, which means responder bias may have influenced our results. The sample size was small and precluded our ability to perform multivariable logistic regression. Generalizability is limited given that this population was primarily Caucasian and from a single institution.

## Conclusion

In summary, 80% of women choose to have another VD after one complicated by an OASIS and 90% have little to no regret with their decision. Increased maternal age and fecal incontinence following delivery with an OASIS are associated with increased regret. All women should receive postpartum education and counseling regarding the diagnosis of OASIS and future implications. Ideally, these conversations would occur in the timeframe of routine postpartum care after the woman has had adequate recovery time from her delivery. Employing shared decision-making regarding subsequent route of delivery following OASIS may minimize decisional regret.

## Data Availability

The datasets generated and/or analyzed during the current study are not publicly available to protect patient privacy but are available from the corresponding author on reasonable request.

## Abbreviations

CD: Cesarean delivery
DRS: Decision Regret Scale
MHHAD: Michigan Healthy Healing After Delivery
OASIS: Obstetrical anal sphincter injury
VD: Vaginal delivery
